# Supplementary material for: In Vitro Anticancer Properties of Novel Bis-Triazoles
Source: Curr Issues Mol Biol. 2022 Dec 29;45(1):175–96. doi: 10.3390/cimb45010014 (PMC9858002; doi:10.3390/cimb45010014)
Supplement: Supplementary file 1 [file cimb-45-00014-s001.zip › Figure S1.pdf]

National Cancer Institute Developmental Therapeutics Program Mean Graphs	NSC :778438/1	Units :Molar	SSPL :0Z4Y	EXP. ID :1405RS75
	Report Date :July 07, 2014		Test Date :May 05, 2014	

Panel/Cell Line	Log_{10}GI50	Log_{10}TGI	Log_{10}LC50
**Leukemia**			
CCRF-CEM	-6.60	> -4.00	> -4.00
HL-60(TB)	-6.65	-6.19	> -4.00
K-562	-6.22	-4.84	> -4.00
MOLT-4	-6.65	-6.22	> -4.00
RPMI-8226	-6.20	-5.41	> -4.00
SR	-6.34	-5.27	> -4.00
**Non-Small Cell Lung Cancer**			
A549/ATCC	-5.84	-5.50	-5.15
EKVX	-5.81	-5.52	-5.22
HOP-62	-5.81	-5.54	-5.26
HOP-92	-6.37	-5.86	-5.23
NCI-H226	-5.81	-5.51	-5.20
NCI-H23	-6.57	-6.10	-4.61
NCI-H322M	-6.54	-5.98	-5.46
NCI-H460	-6.46	-5.86	-5.20
NCI-H522	-6.71	-6.32	-5.32
**Colon Cancer**			
COLO 205	-6.76	-6.48	-6.19
HCC-2998	-6.50	-5.95	-5.35
HCT-116	-6.55	-5.94	-5.45
HCT-15	-4.64	> -4.00	> -4.00
HT29	-6.49	-6.05	-4.47
KM12	-6.26	-5.66	-5.19
SW-620	-6.39	-5.76	-5.13
**CNS Cancer**			
SF-268	-6.49	-5.84	-5.16
SF-295	-5.87	-5.57	-5.26
SF-539	-6.70	-6.38	-6.06
SNB-19	-6.74	-6.43	-6.12
SNB-75	-6.75	-6.38	-5.99
U251	-6.76	-6.46	-6.16
**Melanoma**			
LOX IMVI	-6.73	-6.31	-5.58
MALME-3M	-6.96	-6.58	-6.20
M14	-5.80	-5.47	-5.14
MDA-MB-435	-6.75	-6.49	-6.22
SK-MEL-2	-6.66	-6.26	-5.06
SK-MEL-28	-6.04	-5.61	-5.20
SK-MEL-5	-6.64	-6.15	-5.57
UACC-257	-6.15	-5.62	-5.15
UACC-62	-5.98	-5.59	-5.20
**Ovarian Cancer**			
IGROV1	-6.63	-6.16	-5.42
OVCAR-3	-6.58	-6.12	-5.13
OVCAR-4	-6.88	-6.59	-6.29
OVCAR-5	-6.23	-5.60	-5.04
OVCAR-8	-5.81	-5.44	
NCI/ADR-RES	> -4.00	> -4.00	> -4.00
SK-OV-3	-5.70	-5.27	-4.43
**Renal Cancer**			
786-0	-4.62	> -4.00	> -4.00
A498	-5.79	-5.22	-4.59
ACHN	-5.02	-4.63	-4.26
CAKI-1	> -4.00	> -4.00	> -4.00
RXF 393	-5.44	-4.82	-4.26
SN12C	-6.73	-6.43	-6.13
TK-10	-4.95	-4.52	-4.09
UO-31	-4.77	-4.42	-4.07
**Prostate Cancer**			
PC-3	-5.62	-5.12	> -4.00
DU-145	-5.99	-5.65	-5.32
**Breast Cancer**			
MCF7	-6.84	-6.45	-6.05
MDA-MB-231/ATCC	-6.60	-4.99	-4.21
HS 578T	-5.54	-5.04	> -4.00
BT-549	-5.76	-5.50	-5.24
MDA-MB-468	-6.76	-6.42	-6.07
_MID	-6.13	-5.62	-5.02
Delta	0.83	0.97	1.27
Range	2.96	2.59	2.29

Axis (GI50 / TGI / LC50): +3 +2 +1 0 -1 -2 -3
