# Supplementary material for: In Vitro Anticancer Properties of Novel Bis-Triazoles
Source: Curr Issues Mol Biol. 2022 Dec 29;45(1):175–96. doi: 10.3390/cimb45010014 (PMC9858002; doi:10.3390/cimb45010014)
Supplement: Supplementary file 1 [file cimb-45-00014-s001.zip › Figure S2.pdf]

Supplementary Materials (Fig. S2) – Cell Cycle

Flow cytometric cell cycle analysis of human melanoma MDA-MB-435 cells, treated with **MS47** and **MS49** of 0.5 × GI_{50}, 1 × GI_{50} and 2 × GI_{50} concentrations for **(A)** 24 h **(B)** 48 h and **(C)** 72 h compared to the control cells and stained with propidium iodide (PI).

A)

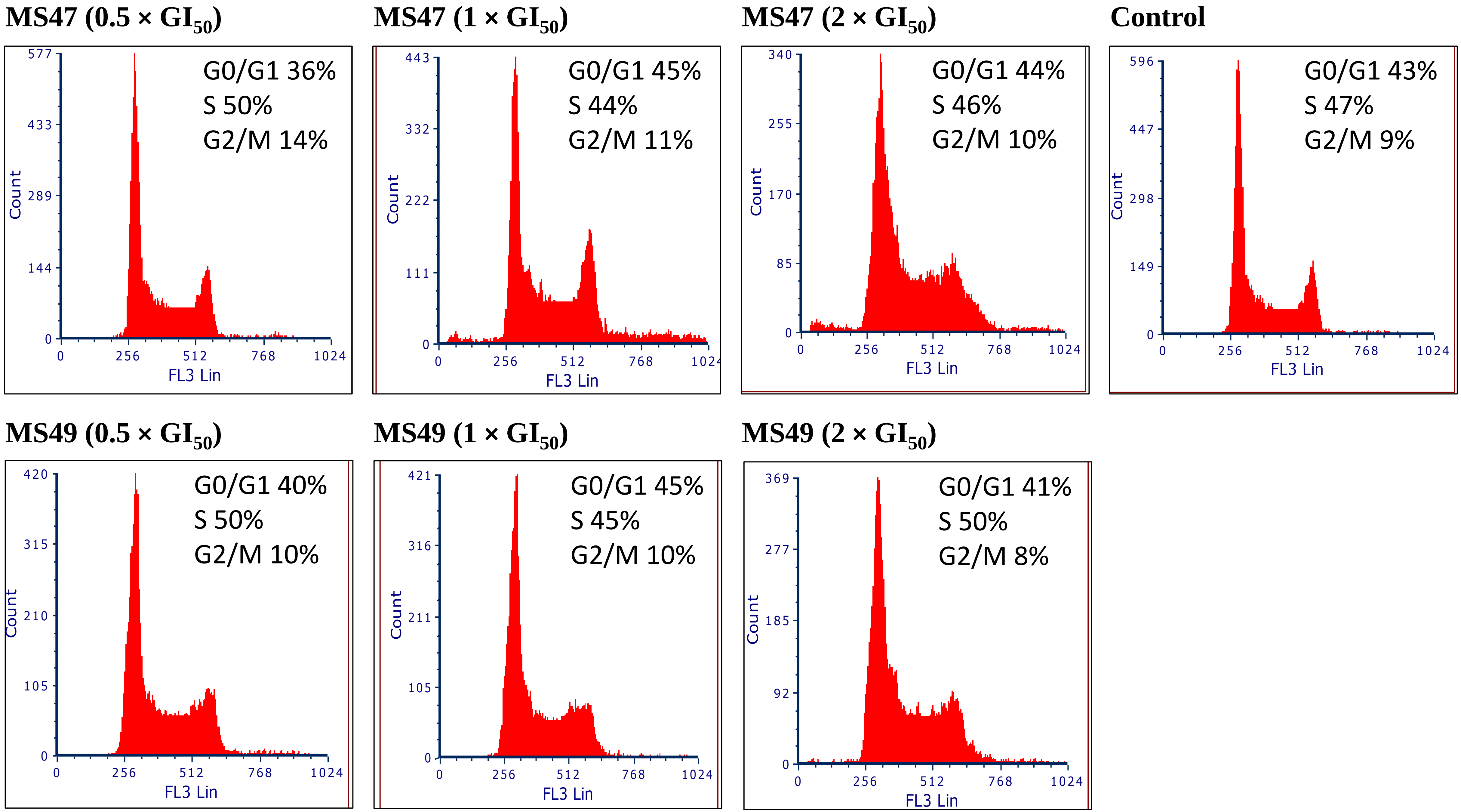

MS47 (0.5 × GI50)
G0/G1 36%
S 50%
G2/M 14%
MS47 (1 × GI50)
G0/G1 45%
S 44%
G2/M 11%
MS47 (2 × GI50)
G0/G1 44%
S 46%
G2/M 10%
Control
G0/G1 43%
S 47%
G2/M 9%
MS49 (0.5 × GI50)
G0/G1 40%
S 50%
G2/M 10%
MS49 (1 × GI50)
G0/G1 45%
S 45%
G2/M 10%
MS49 (2 × GI50)
G0/G1 41%
S 50%
G2/M 8%
Count
FL3 Lin

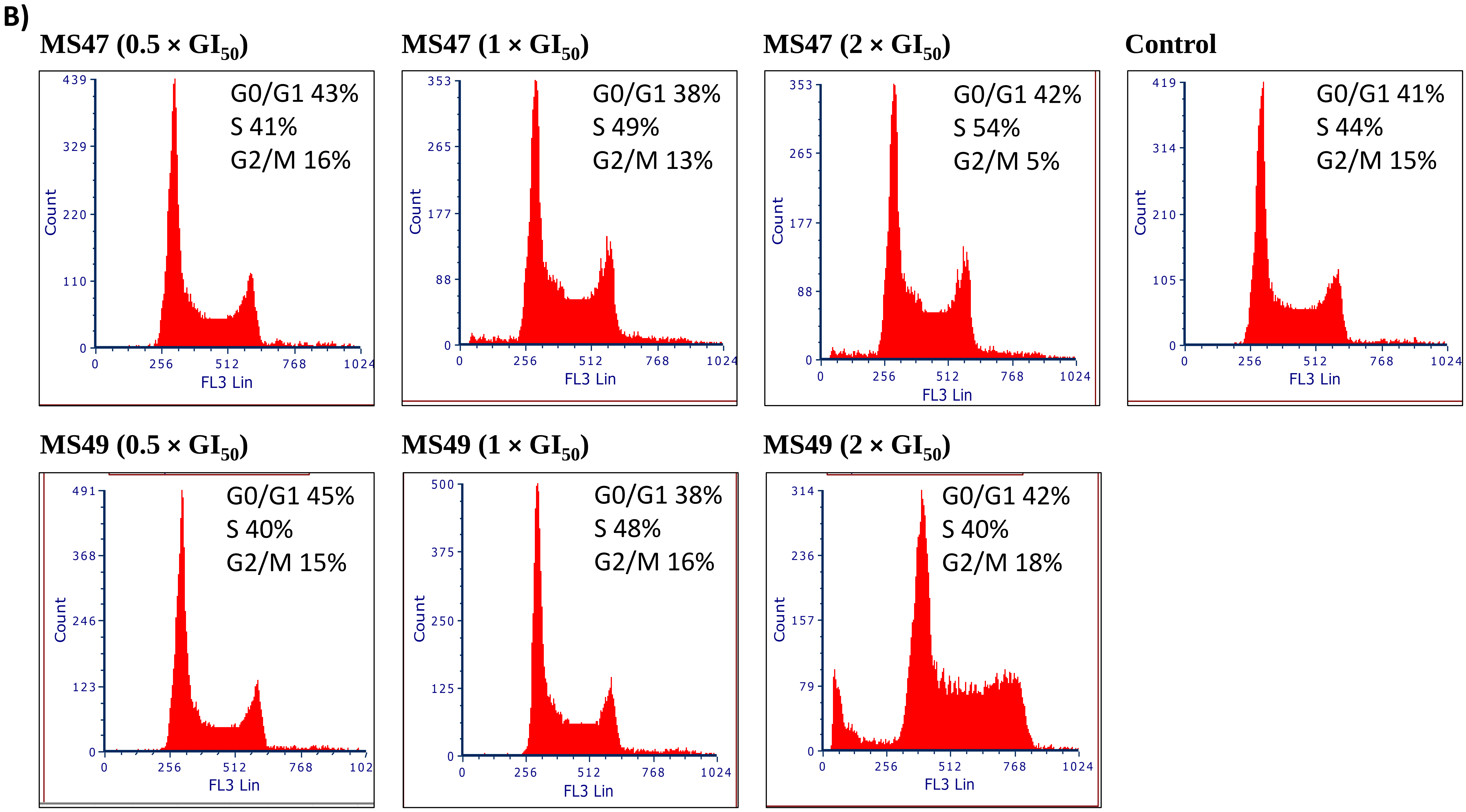
B)
MS47 (0.5 × GI50)
G0/G1 43%
S 41%
G2/M 16%
MS47 (1 × GI50)
G0/G1 38%
S 49%
G2/M 13%
MS47 (2 × GI50)
G0/G1 42%
S 54%
G2/M 5%
Control
G0/G1 41%
S 44%
G2/M 15%
MS49 (0.5 × GI50)
G0/G1 45%
S 40%
G2/M 15%
MS49 (1 × GI50)
G0/G1 38%
S 48%
G2/M 16%
MS49 (2 × GI50)
G0/G1 42%
S 40%
G2/M 18%
Count
FL3 Lin

c)

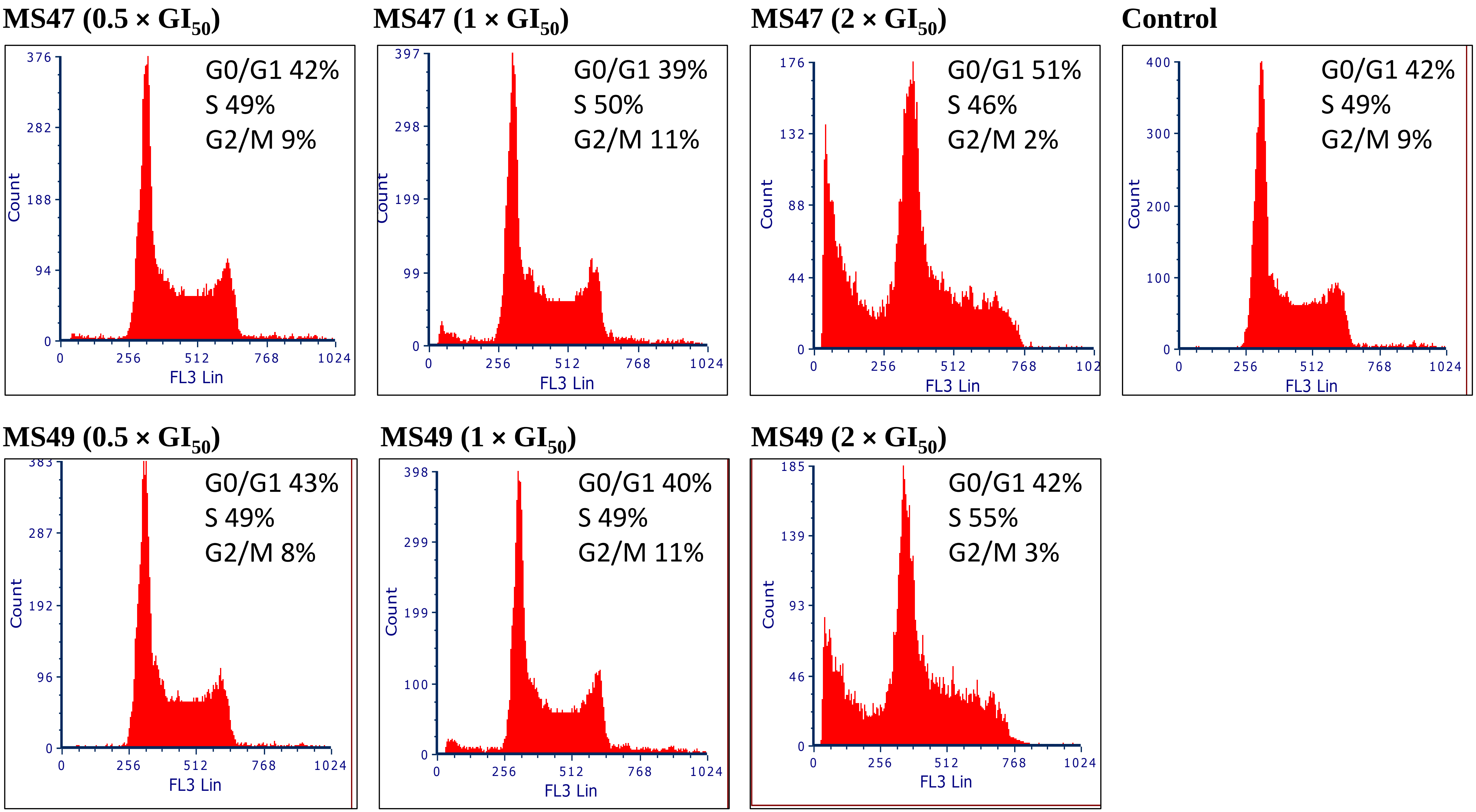
MS47 (0.5 × GI50)
G0/G1 42%
S 49%
G2/M 9%
MS47 (1 × GI50)
G0/G1 39%
S 50%
G2/M 11%
MS47 (2 × GI50)
G0/G1 51%
S 46%
G2/M 2%
Control
G0/G1 42%
S 49%
G2/M 9%
MS49 (0.5 × GI50)
G0/G1 43%
S 49%
G2/M 8%
MS49 (1 × GI50)
G0/G1 40%
S 49%
G2/M 11%
MS49 (2 × GI50)
G0/G1 42%
S 55%
G2/M 3%
Count
FL3 Lin
0
256
512
768
1024

A)

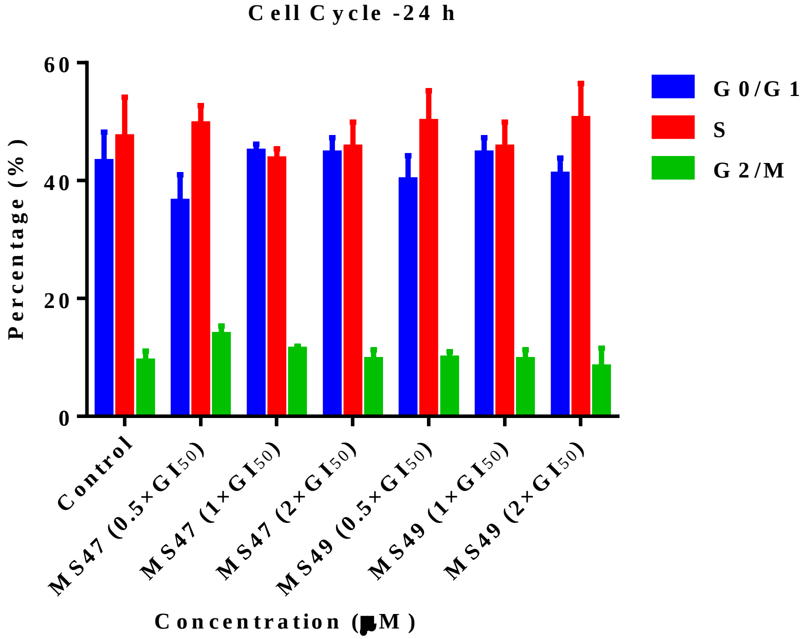
Cell Cycle -24 h
Percentage (%)
0
20
40
60
G0/G1
S
G2/M
Control
MS47 (0.5×GI50)
MS47 (1×GI50)
MS47 (2×GI50)
MS49 (0.5×GI50)
MS49 (1×GI50)
MS49 (2×GI50)
Concentration (µM)

B)

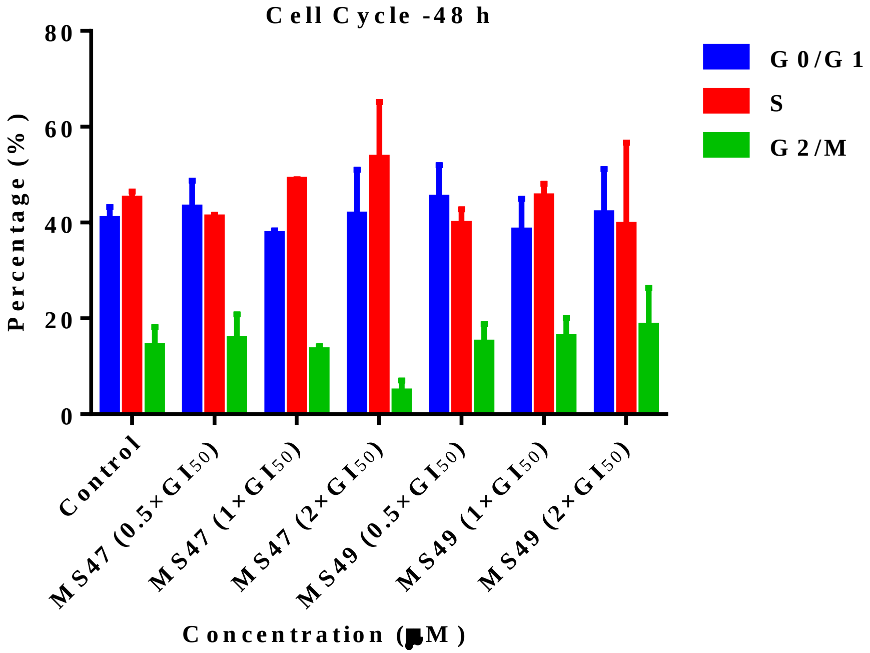
Cell Cycle -48 h
Percentage (%)
0
20
40
60
80
G0/G1
S
G2/M
Control
MS47 (0.5×GI50)
MS47 (1×GI50)
MS47 (2×GI50)
MS49 (0.5×GI50)
MS49 (1×GI50)
MS49 (2×GI50)
Concentration (µM)

C)

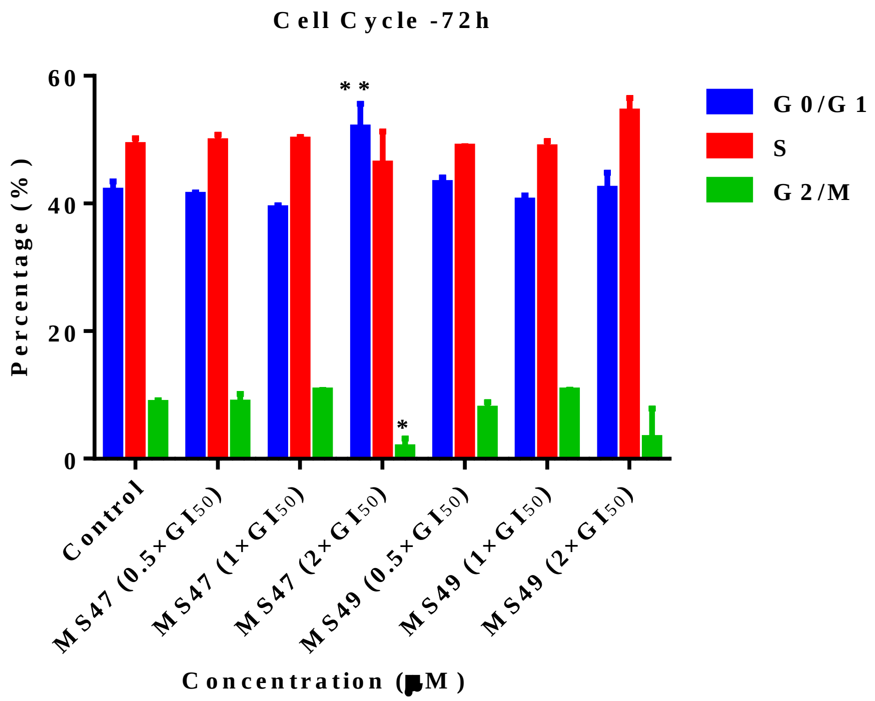

Cell Cycle -72h
Percentage (%)
60
40
20
0
**
*
G0/G1
S
G2/M
Control
MS47 (0.5×GI50)
MS47 (1×GI50)
MS47 (2×GI50)
MS49 (0.5×GI50)
MS49 (1×GI50)
MS49 (2×GI50)
Concentration (µM)
