# Supplementary material for: In Vitro Anticancer Properties of Novel Bis-Triazoles
Source: Curr Issues Mol Biol. 2022 Dec 29;45(1):175–96. doi: 10.3390/cimb45010014 (PMC9858002; doi:10.3390/cimb45010014)
Supplement: Supplementary file 1 [file cimb-45-00014-s001.zip › Figure S3.pdf]

Supplementary Materials (Fig. S3) – Apoptosis

Flow cytometric analysis of cell death mechanism (Apoptosis/Necrosis) in human melanoma MDA-MB435 cells, treated with **MS47** and **MS49** of 0.5 × GI_{50}, 1 × GI_{50} and 2 × GI_{50} concentrations compared to the untreated cells (control group) and stained with FITC-conjugated annexin V and propidium iodide (PI) for **(A)** 24 h **(B)** 48 h and **(C)** 72 h.

A)

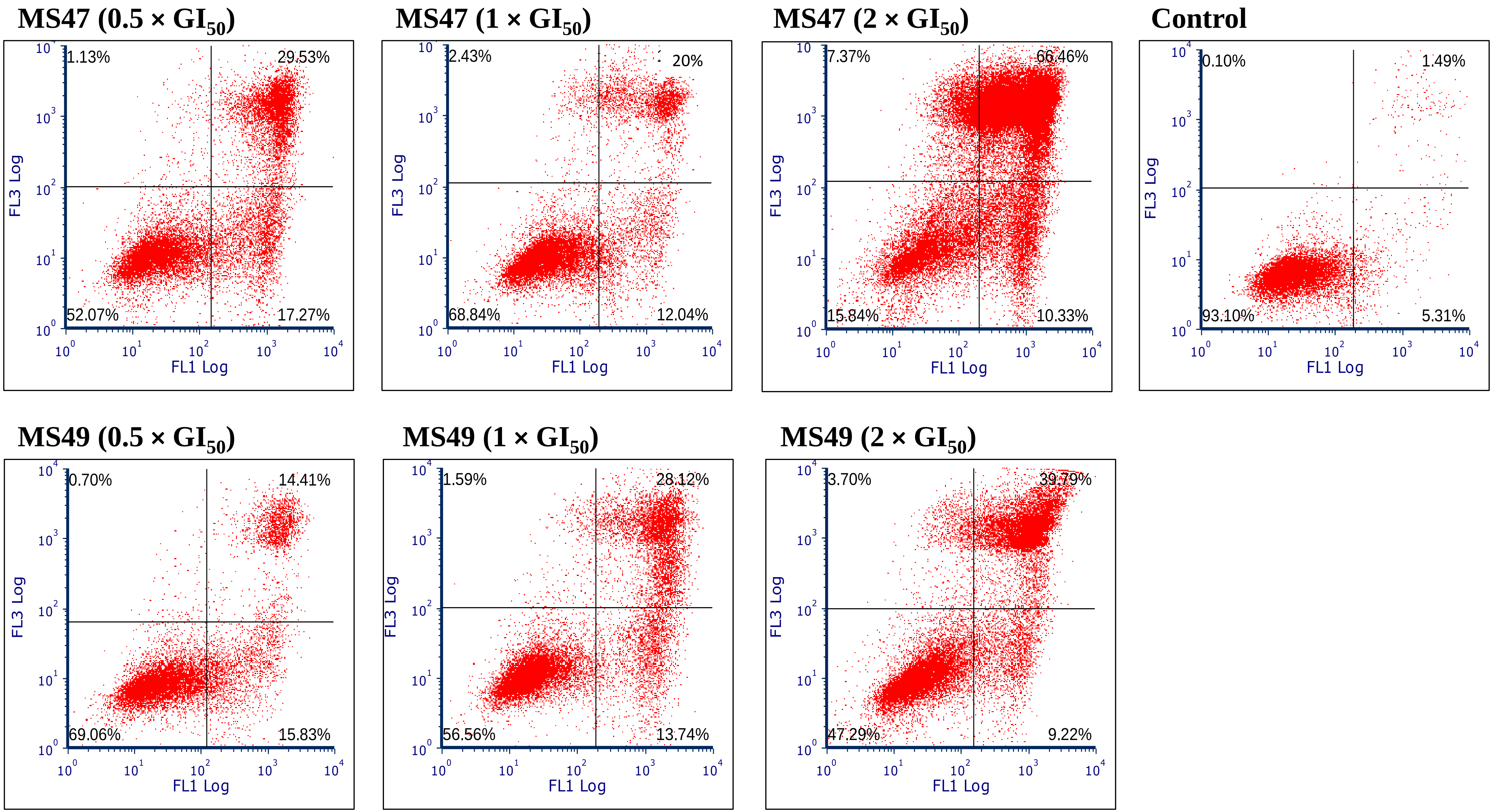

MS47 (0.5 × GI50)
1.13%
29.53%
52.07%
17.27%
MS47 (1 × GI50)
2.43%
20%
68.84%
12.04%
MS47 (2 × GI50)
7.37%
66.46%
15.84%
10.33%
Control
0.10%
1.49%
93.10%
5.31%
MS49 (0.5 × GI50)
0.70%
14.41%
69.06%
15.83%
MS49 (1 × GI50)
1.59%
28.12%
56.56%
13.74%
MS49 (2 × GI50)
3.70%
39.79%
47.29%
9.22%
FL3 Log
FL1 Log

B)

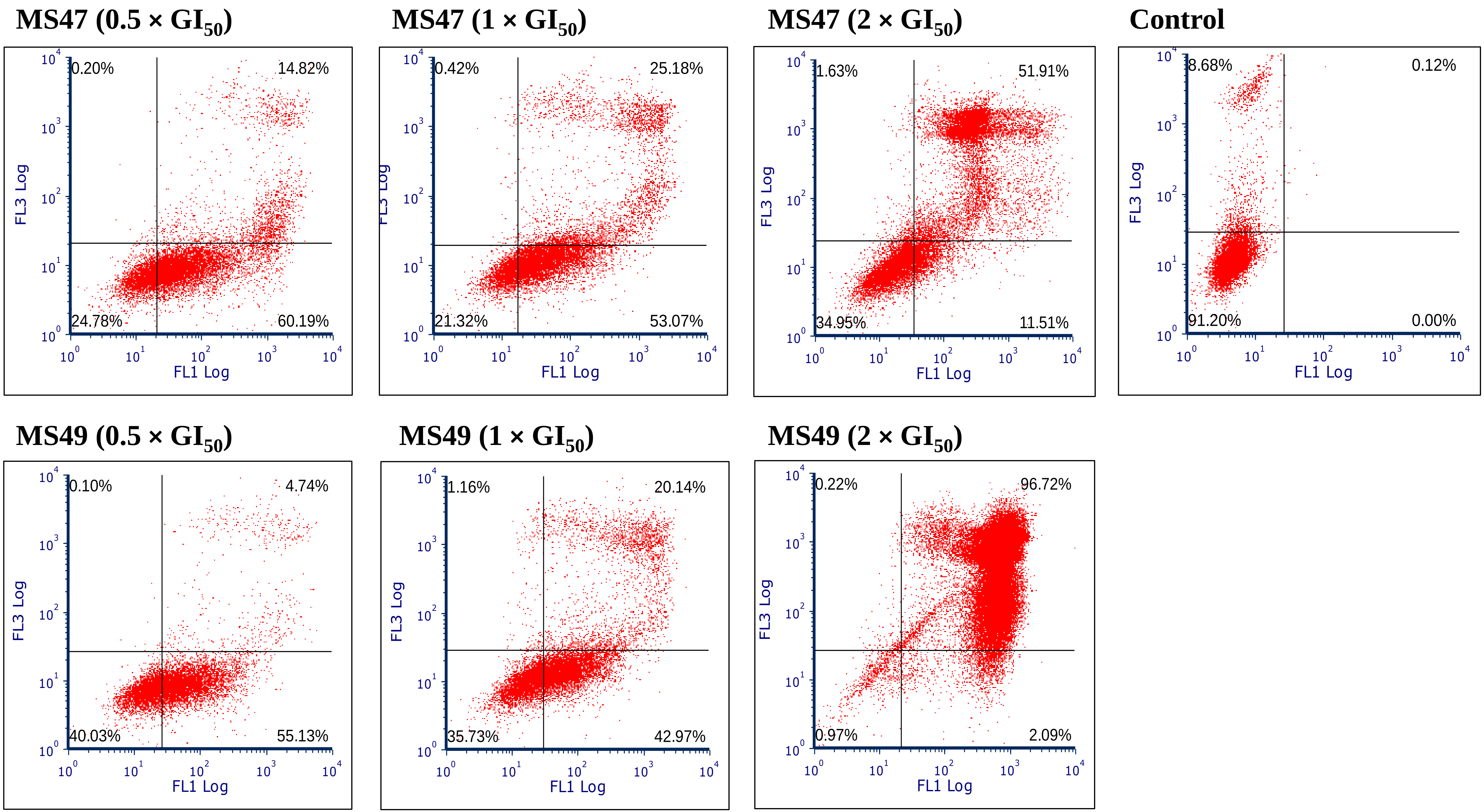

MS47 (0.5 × GI50)
0.20%
14.82%
24.78%
60.19%
MS47 (1 × GI50)
0.42%
25.18%
21.32%
53.07%
MS47 (2 × GI50)
1.63%
51.91%
34.95%
11.51%
Control
8.68%
0.12%
91.20%
0.00%
MS49 (0.5 × GI50)
0.10%
4.74%
40.03%
55.13%
MS49 (1 × GI50)
1.16%
20.14%
35.73%
42.97%
MS49 (2 × GI50)
0.22%
96.72%
0.97%
2.09%
FL3 Log
FL1 Log

c)

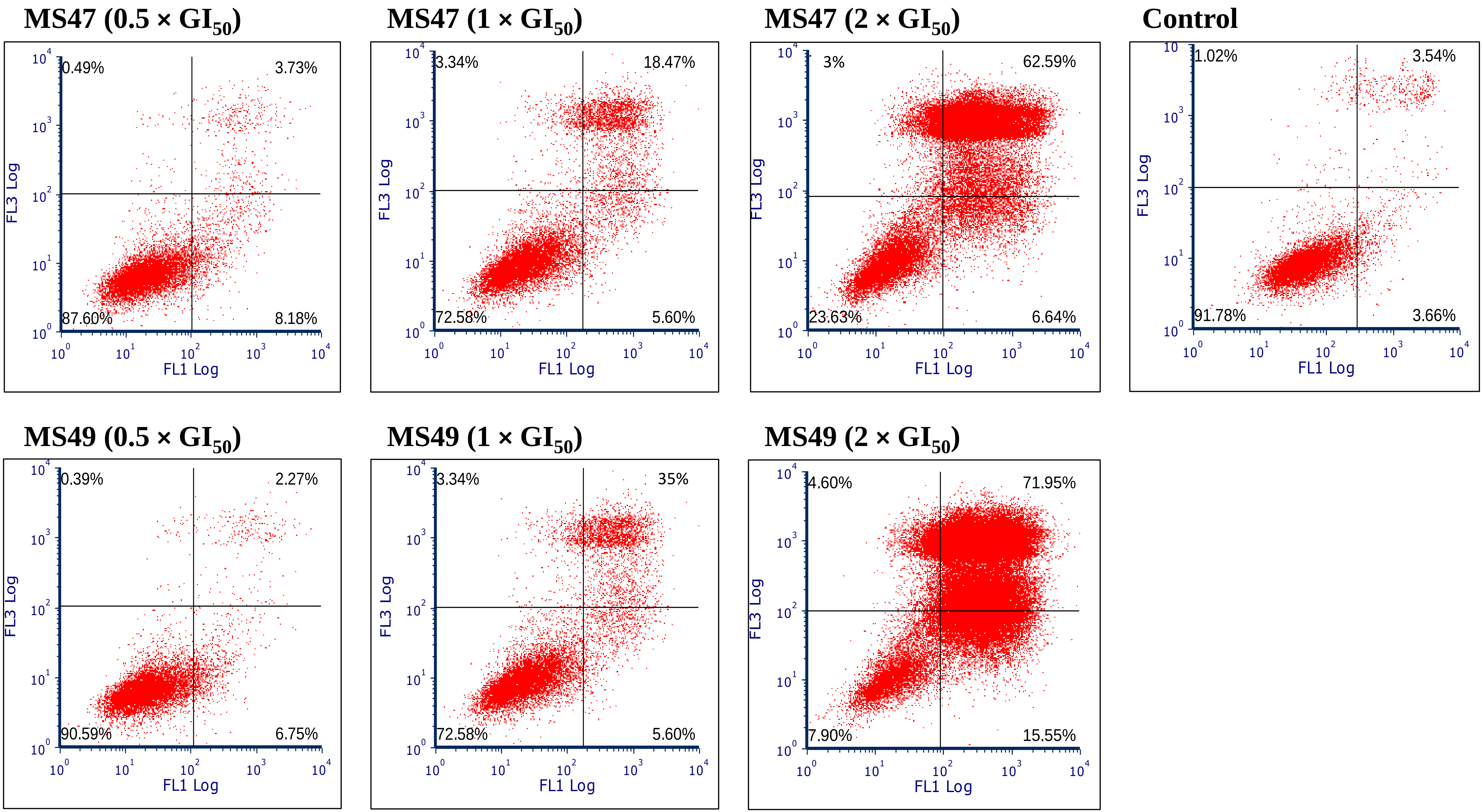
MS47 (0.5 × GI50)
0.49%
3.73%
87.60%
8.18%
MS47 (1 × GI50)
3.34%
18.47%
72.58%
5.60%
MS47 (2 × GI50)
3%
62.59%
23.63%
6.64%
Control
1.02%
3.54%
91.78%
3.66%
MS49 (0.5 × GI50)
0.39%
2.27%
90.59%
6.75%
MS49 (1 × GI50)
3.34%
35%
72.58%
5.60%
MS49 (2 × GI50)
4.60%
71.95%
7.90%
15.55%
FL3 Log
FL1 Log
