# Supplementary figures and images for: In Vitro Anticancer Properties of Novel Bis-Triazoles
Source: Curr Issues Mol Biol. 2022 Dec 29;45(1):175–96. doi: 10.3390/cimb45010014 (PMC9858002; doi:10.3390/cimb45010014)

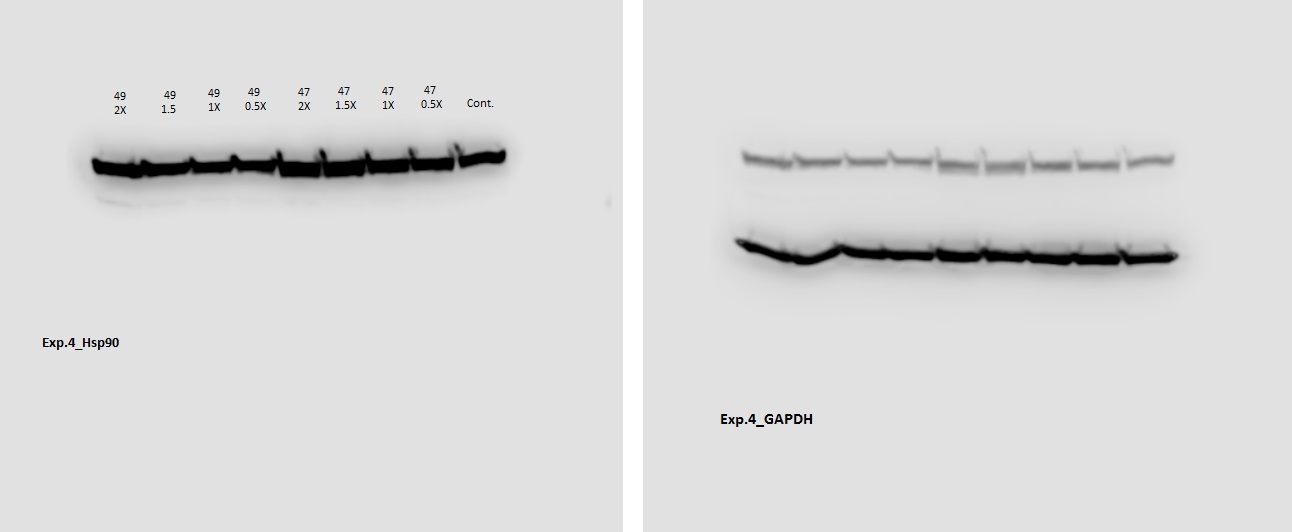

Supplement: Supplementary file 1 [file cimb-45-00014-s001.zip › Figure S4 (A).jpg]

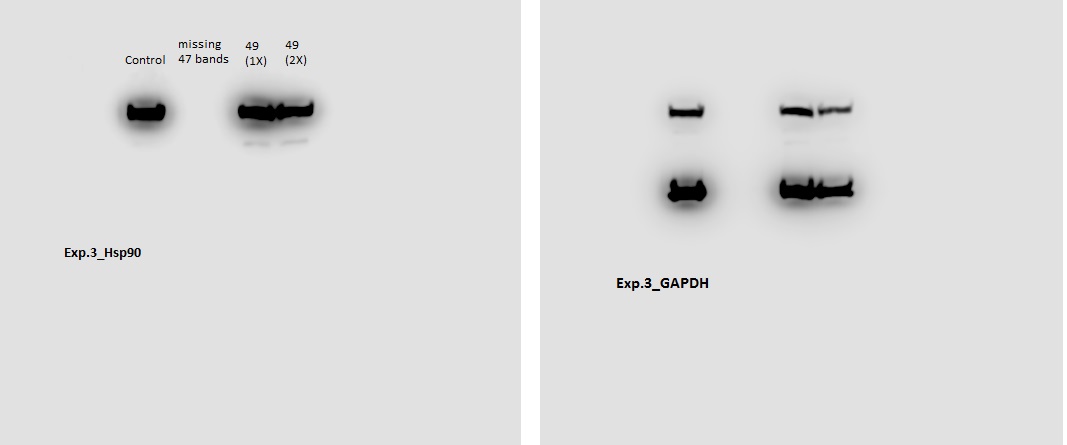

Supplement: Supplementary file 1 [file cimb-45-00014-s001.zip › Figure S4 (B).jpg]
